# *Salicornia europaea* L. Functional Traits Indicate Its Optimum Growth

**DOI:** 10.3390/plants11081051

**Published:** 2022-04-12

**Authors:** Stefany Cárdenas-Pérez, Ahmad Rajabi Dehnavi, Karol Leszczyński, Sandra Lubińska-Mielińska, Agnieszka Ludwiczak, Agnieszka Piernik

**Affiliations:** 1 Department of Geobotany and Landscape Planning, Faculty of Biological and Veterinary Sciences, Nicolaus Copernicus University in Torun, Lwowska 1, 87-100 Torun, Poland; cardenasperez@umk.pl (S.C.-P.); ahmad.rajabi.dehnavi@gmail.com (A.R.D.); karol.leszczynski96@gmail.com (K.L.); sanlub@doktorant.umk.pl (S.L.-M.); agnieszka.lud@umk.pl (A.L.); 2 Department of Agronomy and Plant Breeding, College of Agriculture, Isfahan University of Technology, Isfahan 84156-83111, Iran

**Keywords:** halophytes, salinity, morphology, anatomy, catalase, peroxidase, hydrogen peroxide, chlorophyll content

## Abstract

*Salicornia europaea* L. grows in areas periodically flooded by salty or brackish water. It has potential economic value, because it can be used as food, forage, or biofuel, and has potential in pharmaceuticals and cosmetics. Increasing interest in *S. europaea* is due to its extreme salt tolerance and well growth in marginal saline soils. However, the variation in its functional traits in response to environmental conditions is still poorly studied. There are still questions regarding the optimal level of salinity for different traits. Therefore, we worked to address the question if *S. europaea* traits from different scales are controlled by salinity level. Based on performed pot experiment, we found that almost all traits are salinity dependent but affected in different ways. We demonstrated that morphological, biomass, and anatomical properties indicate optimum growth between 200 and 400 mM NaCl and growth limitations at 0, 800, and 1000 mM NaCl. Moreover, we found the most affected traits which include photosynthetic pigments and protein content, plant surface area, peroxidase activity, and anatomic traits related to cell shape. Our results significantly expanded the knowledge about *S. europaea* functional traits variation in response to salinity, which can be important for discovering regulating processes and for possible future agricultural applications.

## 1. Introduction

*Salicornia europaea* L. belongs to the *Amaranthaceae* family (formerly *Chenopodiaceae*), subfam. *Salicornioideae*. The genus *Salicornia* is widespread in temperate and subtropical regions of the Northern Hemisphere but absent in South America and Australia [1]. Presence in habitats with changing seasonal and even daily dynamics has led to high physiological plasticity in plants [2]. This results in phenotypic variability and problems with establishing acceptable systematics [3]. Despite the high phenotypic variability, several common features can be distinguished [4,5]. *S. europaea* has an erect, highly branched stem. Secondary shoots are formed on the primary cylindrical shoots. The plant has strongly reduced leaves, and the assimilation area is located in the shoots. It is green most of its life, but at the end of life cycle, the stems turn red due to chlorophyll destruction which reduces photosynthesis and finally affects nutrient loss, biomass, and hydric balance [6]. Branches have spikes consisting of three flowers, one main and two laterals. Seeds are small, dark, ellipsoidal, and characterized by heteromorphism, i.e., color, shape, and size variability [7].

*S. europaea* grows in areas periodically flooded by salty or brackish water [8]. In Central Europe this species has been recognized as *Salicornia ramosissima* J. Woods (=*S. herbacea* L.) [9]. It has potential economic value because it is edible, either raw or cooked [10]. In addition, it can be used as a forage for animals or a biofuel and has potential in pharmaceuticals and cosmetics [11,12]. It also has a high fatty acid content in its seeds, which increases its nutritional value [13]. Moreover, *S. europaea* ash can be used to produce glass and soap [14]. The healing properties should also be mentioned because *S. europaea* is rich in tungsten acids, quercetin, and isorhamnetin, which have anti-inflammatory and antioxidant properties. It also contains polysaccharides that play a role in the treatment of constipation, obesity, diabetes, and cancer [14,15].

This species belongs to extreme halophytes [16], which is a group of species strongly adapted to saline environments. They evolved some specific mechanisms to cope with saline environment like reduction of the Na^+^ levels, compartmentalization, and excretion of sodium ions [17,18]. The first *S. europaea* mechanism to overcome high Na^+^ concentrations is the water storage in the parenchyma, which dilutes the accumulated salts and contributes to maintaining cellular turgor. This allows the plant to cope efficiently with high salinity [16]. However, reduction of growth of this species have been reported at high salinities [19,20]. The adaptation to different levels of salinity can affect water storage intensity and therefore some anatomical properties, e.g., the size and shape of stem-cortex cells [21]. Moreover, in the salinity gradient plants induce some physiological responses related to osmotic adjustment as proline accumulation, and increasing antioxidant enzyme activities, e.g., catalase (CAT) and peroxidase (POD) [19,22]. Salinity can also affect photosynthetic activity by changes in chlorophyll and carotenoids content [23,24] and different proteins content, playing important roles for plant salt tolerance ability [25].

Increasing interest in *S. europaea* is due to its extreme salt tolerance over 1000 mM NaCl [8]. Results of recent studies at the International Center for Biosaline Agriculture (ICBA) in the United Arab Emirates (UAE) show that some varieties of *S. europaea* cultivated with good agronomic practices grow well in marginal soils and can be economically viable [8]. As an obligatory halophyte, it is believed by definition to be able to complete its life cycle in a salt concentration of around 200 mM NaCl (ca. 20 dSm^−1^) or more under conditions like those that might be encountered in the natural environment [17]. Although, halophytes are species that can live and reproduce successfully under salt stress; it is still not so clear if they need salt for development. Regarding *S. europaea*, Snow and Vince [26] reported better growth of this species outside their home zone in salt marsh habitats and its presence at high salinity because of low competitive ability with other species. It was partly confirmed by Piernik [27] who found, under field conditions, the good growth of this species at lower salinity than in its home vegetation zone. Mucolo et al. [2] found that the very high final germination in distilled water (control) suggests that these taxa do not necessarily have a physiological requirement for salt to germinate.

There are also still few studies reporting or focusing on *S. europaea*’s optimum growth assessment. Lv et al. [28] reported *S. europaea* optimal growth and photosynthetic rates at 200–400 mM NaCl. Araus et al. [8] reported that the best irrigation regime in terms of biomass and seed yield involved brackish water of 25 dSm^−1^. Similar results were obtained by Singh et al. [29], i.e., a notable amount of biomass for *S. ramosissima* using artificial seawater containing 257 mM NaCl. Except for biomass and seeds yield, there is still a lack of knowledge of *S. europaea*’s morphological and anatomical trait adaptations to different salinity levels and their optimum growth [4,5,16,21,30].

Plant functional traits are defined as any morphological, anatomical, physiological, and phenological plant characteristics affecting overall plant fitness through their influence on survival, growth, and reproduction [31]. They determine how primary producers respond, among others, to environmental factors, affect trophic levels, influence ecosystem processes and services, and provide a link from species richness to ecosystem functional diversity [32]. Plant functional trait data, in the form of species-level trait measurements, are increasingly accessible from large databases [33,34]. However, the variation in functional traits in response to environmental conditions is still poorly understood [35,36]. Variability of functional traits is important because it can play a role in adaptive and non-adaptive processes under changing environments [37,38,39]. In case of *S. europaea*, few studies report physiological traits responses [19,20,22,40]. The biochemical parameters, although useful, are black boxes in terms of anatomical and structural changes, which are not directly visualized. This is currently a gap in the literature for this species. To our knowledge, there are no comprehensive studies considering traits in the context of the adaptation and optimum growth of *S. europaea* in the salinity gradient. The level of salinity is still under question, which can be considered as optimal for *S. europaea* trait development. Moreover, it is still unknown which *S. europaea* functional traits are the most affected by salinity.

Therefore, to fill this gap in the knowledge the overarching question, we worked to address whether *S. europaea* traits are from different scales controlled by salinity level. To answer this question, we performed complex research on the morphological, anatomical, and physiological traits at different salinity levels. For morphological and anatomical assessments, we applied a novel image analysis method [5]. To present a complex example of the plant trait functional linkage, we applied similarity analysis between different salt treatments [41]. We also selected the most affected by salinity functional traits by the means of discriminant analysis [42]. We hypothesized that: (a) salinity affects plant morphological, anatomical, and physiological responses in different ways, and (b) plant trait responses can indicate optimum growth in the salinity gradient. Understanding complex mechanisms of salt stress adaptation of *S. europaea*, an extreme halophyte species, is possible only based on traits from different functional levels. The determination of key functional traits in salinity adaptations and *S. europaea* optimum growth is also important because of the possible future agricultural application perspectives.

## 2. Results

### 2.1. Growth Responses to Different Salinity Levels

**Figure 1:**
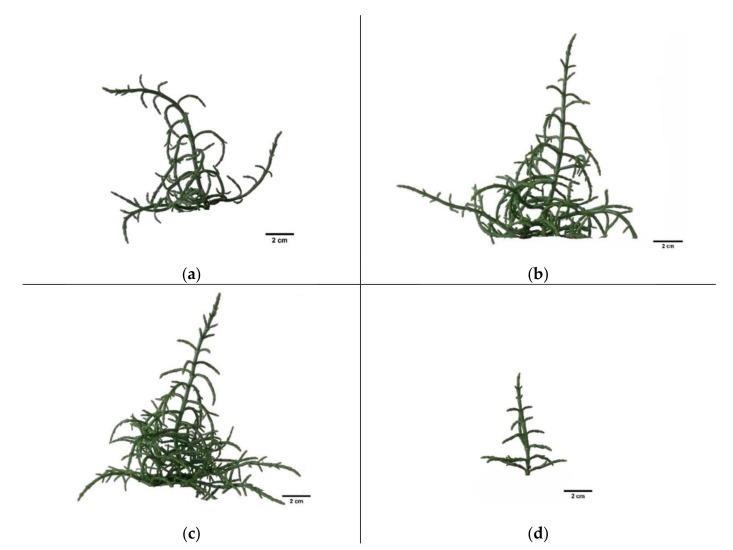
Images of *Salicornia europaea* L. grown in different NaCl concentrations: (**a**) 0 mM; (**b**) 400 mM; (**c**) 800 mM; (**d**) 1000 mM.

**Figure 2:**
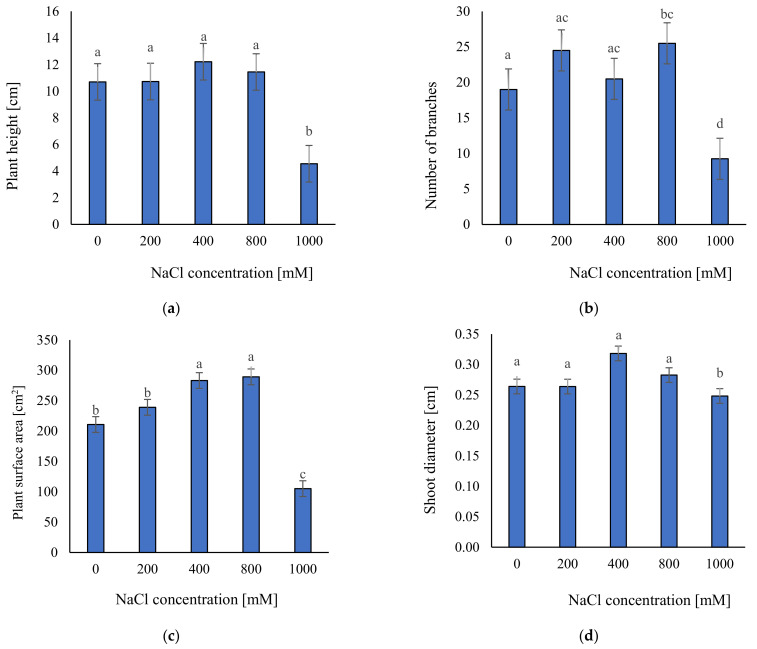
The average parameters of: (**a**) height; (**b**) number of branches; (**c**); surface area; (**d**) shoot diameter of the analyzed plants; ±SD (standard deviation) in the tested samples. ANOVA *p* < 0.001, significant differences based on post hoc Tukey’s test are marked with different letters.

To investigate the morphological trait responses to salt stress, we measured plant height, number of branches, plant surface area, and shoot diameter. We found differences in the morphological features dependent on the salt concentrations (Figure 1 and Figure 2). Plants which grew under extreme salinity (1000 mM NaCl) were smaller than those grown in other salt treatments. They had the smallest plant surface area (ca. 105 cm^2^) and were significantly shorter (ca. 4.6 cm) and thinner (diameter ca. 0.248 cm) than the others (Figure 2). Moreover, they had the lowest number of branches (ca. 9). There were not significant differences in plant height and shoot diameter between 0, 200, 400, and 800 mM NaCl treatments (Figure 2a,d). The plant surface area was higher in 400 (283 cm^2^) and 800 mM NaCl (ca. 289 cm^2^) (Figure 2c). The highest number of branches (ca. 26) was noted at 800 mM, but there were no significant differences between 800, 400, and 200 mM treatments (Figure 2b). Therefore, the relationship between this trait and salinity is not so clear. 

**Figure 3:**
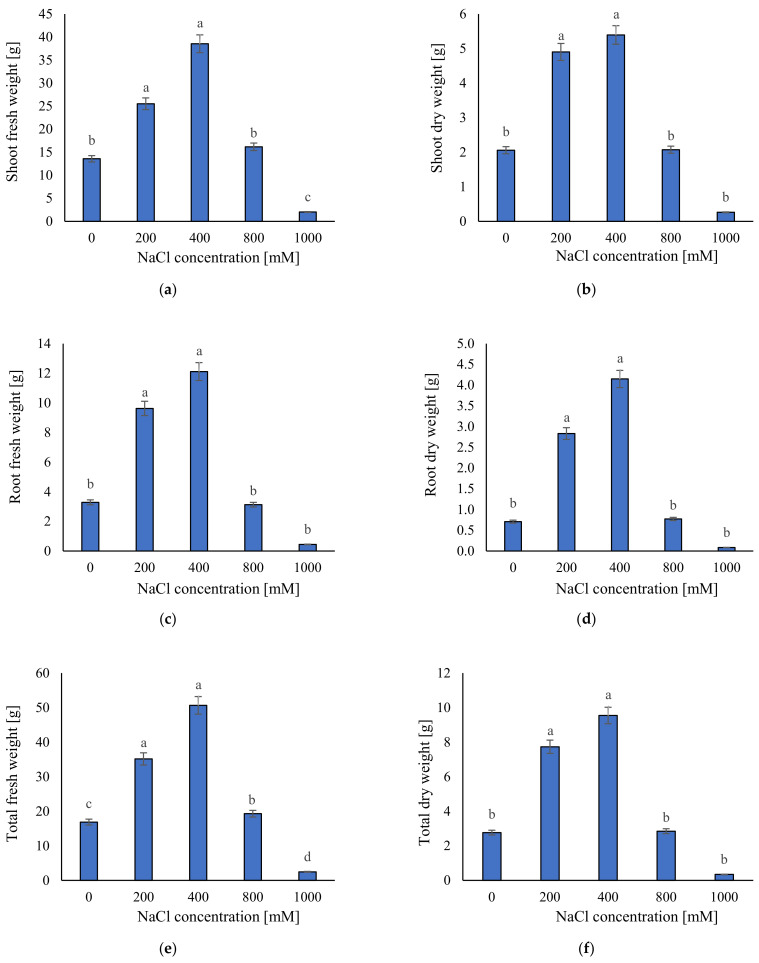
The average parameters of: (**a**) shoot fresh weight; (**b**) shoot dry weight; (**c**) root fresh weight; (**d**) root dry weight; (**e**) total fresh weight; and (**f**) total dry weight in the tested plants; ±SD in the tested samples. ANOVA *p* < 0.001, significant differences based on post hoc Tukey’s test are marked with different letters.

For the effect of salinity on biomass accumulation, we measured shoot, root, and total fresh and dry weight. The highest values for fresh and dry weight (SFW 38.5 g, RFW 12.1 g, TFW 50.6 g, SDW 5.4 g, RDW 4.1 g, and TDW 9.5 g) were obtained in 400 mM NaCl, although for all these traits there were no statistically significant differences between 400 and 200 mM NaCl (Figure 3). The lowest values for fresh and dry weights (SFW 2.03 g, RFW 0.44 g, TFW 2.47 g, SDW 0.26 g, RDW 0.08 g, and TDW 0.34 g) were obtained in 1000 mM NaCl. In general, for measurements of fresh and dry weights, there were not significant differences between 0, 800, and 1000 mM NaCl (Figure 3). These results confirm morphological traits assessments, i.e., under 1000 mM NaCl, *S. europaea* cannot grow well. Moreover, growth limitations were detected at 0 and 800 mM NaCl.

### 2.2. Anatomical Responses to Different Salinity Levels

**Figure 4:**
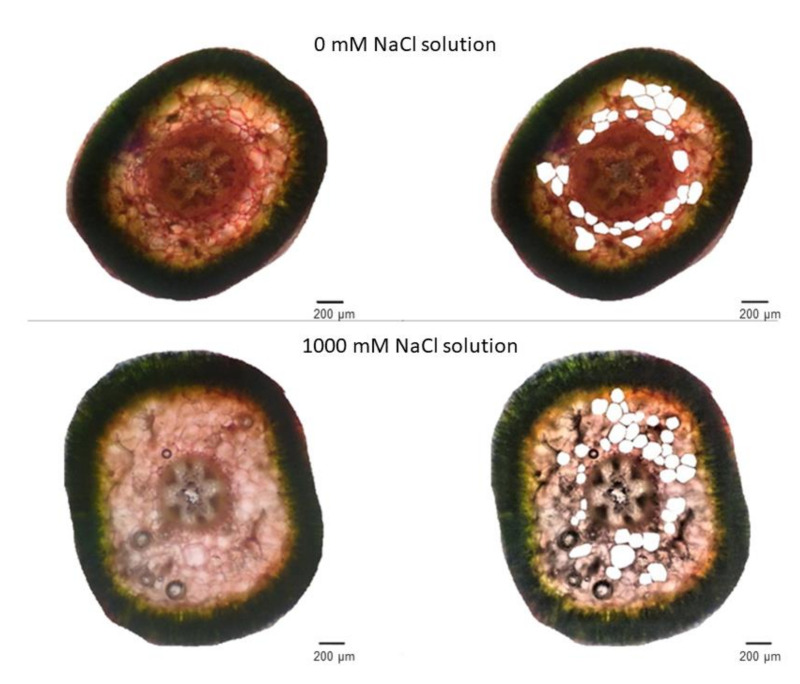
Stem cross-section of *Salicornia europaea* L. from plants treated with 0 and 1000 mM NaCl solutions. At the right side, example cortex cells for measurements by ImageJ software are marked as empty.

**Figure 5:**
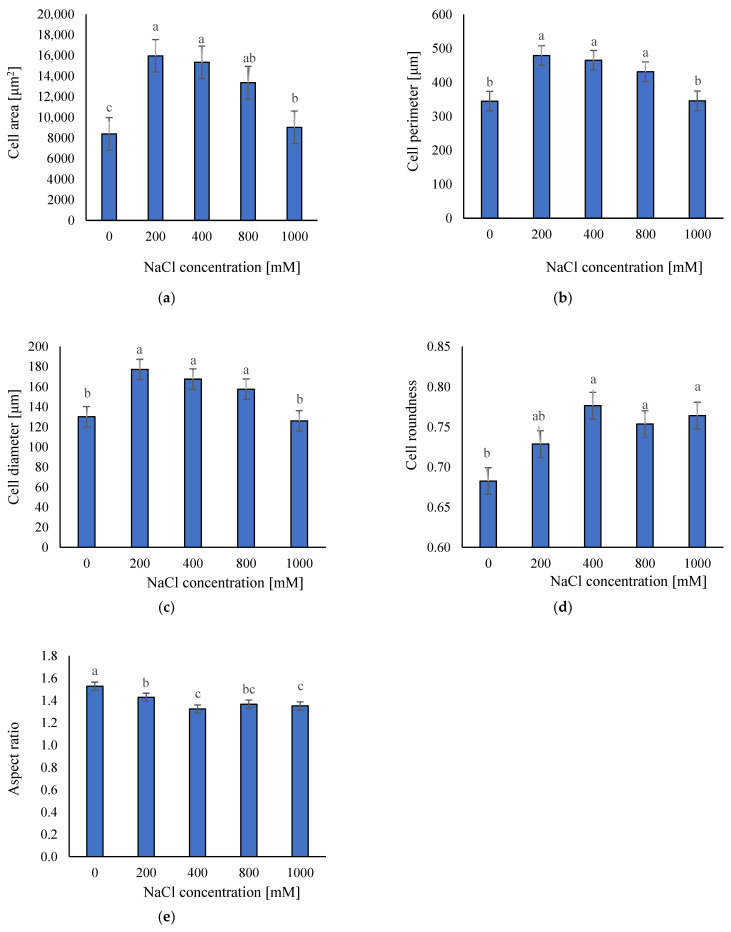
The average parameters of: (**a**) area; (**b**) perimeter; (**c**) diameter; (**d**) roundness; (**e**) aspect ratio of the *Salicornia europaea* L. cells; ±SD in the tested samples. ANOVA *p* < 0.001, significant differences based on post hoc Tukey’s test are marked with different letters.

We based the effect of salinity on anatomical traits on measurements of area, perimeter, diameter, roundness, and aspect ratio of stem-cortex cells (Figure 4). Our results showed that in the control and extremely saline condition (0 and 1000 mM NaCl), the plant cells area, cell perimeter, and cell diameter were lower than in moderate and high saline treatments (200, 400, and 800 mM NaCl) (Figure 5a–c). The plant cells area in the non-saline condition and 1000 mM NaCl was ca. 8394 and 9027 μm^2^ respectively, the plant cell perimeter was ca. 345 and 346 μm, and the cell diameter was ca. 130 and 126 μm. There were not statistically significant differences between 200–800 mM NaCl treatments and cells area ranged between 13.360 and 15.967 μm^2^, cell perimeter between 431 and 479 μm, and cell diameter between 157 and 177 μm (Figure 5a–c). Moreover, we observed that cells of plants grown under non-saline conditions were significantly less spherical (cell roundness of ca. 0.70), and according to the high aspect ratio (1.53) the most elongated (Figure 5d,e).

These results suggested that under non-saline and extreme saline conditions, stem-cortex cells are smaller probably because of growth stress. We did not find statistically significant differences in anatomical traits between plants grown in 200–800 mM NaCl.

### 2.3. Biochemical Response to Different Salinity Levels

#### 2.3.1. Photosynthetic Pigments and Soluble Protein Content

**Figure 6:**
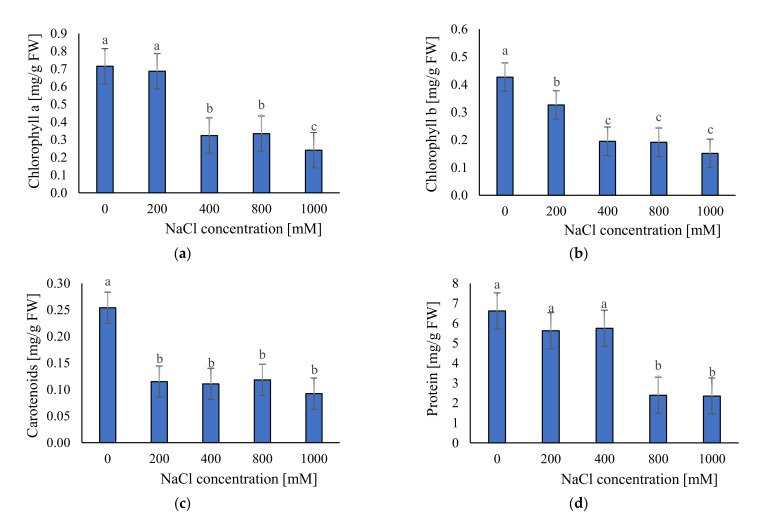
The average concentration of: (**a**) chlorophyll a; (**b**) chlorophyll b; (**c**) carotenoid; and (**d**) soluble protein in the tested plants; ±SD in the tested samples. ANOVA *p* < 0.001, significant differences based on post hoc Tukey’s test are marked with different letters.

Photosynthetic pigments are traits that can affect photosynthetic performance, plant growth, and development. We measured concentrations of chlorophyll a and b and carotenoids. Results demonstrated decreasing photosynthetic pigments content with increasing NaCl concentrations (Figure 6a–c). The highest content was found in control condition (chlorophyll a: 0.715 mg/g FW; chlorophyll b: 0.427 mg/g FW and carotenoids 0.254 mg/g FW). The lowest content was found in 1000 mM NaCl (chlorophyll a: 0.241 mg/g FW; chlorophyll b: 0.151 mg/g FW and carotenoids 0.092 mg/g FW).

Soluble protein content was more stable than pigments because, up to 400 mM NaCl, there were not significant changes in their content (ca. 6.0 mg/g FW) (Figure 6d). However, a significant decrease was observed in 800 (ca. 2.39 mg/g FW) and 1000 mM NaCl (ca. 2.35 mg/g FW) (Figure 6d).

#### 2.3.2. Hydrogen Peroxide (H_2_O_2_) Content

**Figure 7:**
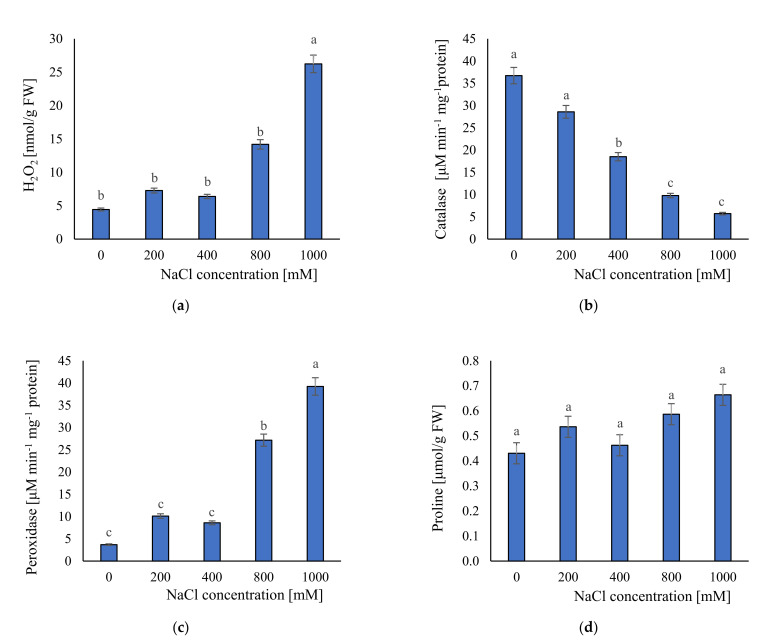
The average parameters of: (**a**) H_2_O_2_ content; (**b**) catalase activity; (**c**) peroxidase activity; and (**d**) proline content in the tested plants; ±SD in the tested samples. ANOVA *p* < 0.001, significant differences based on post hoc Tukey’s test are marked with different letters.

Hydrogen peroxide is a strong ROS (reactive oxygen species) which is overproduced in plant cells under stressful conditions. To investigate how different salinity levels can affect ROS production we measured H_2_O_2_ content. We observed that under 0 (ca. 4.4 nmol/g FW) to 800 mM NaCl (ca. 14.2 nmol/g FW) the level of H_2_O_2_ did not differ significantly (Figure 7a). However, we observed a statistically significant increase of the H_2_O_2_ content in extreme salinity—1000 mM NaCl (ca. 26.2 nmol/g FW). It seems that *S. europaea* has a high tolerance to increasing salinity levels and can prevent H_2_O_2_ overproduction.

#### 2.3.3. Antioxidant Enzymes Activities and Proline Content

Antioxidant enzymes activities and osmolyte accumulation are two of the most important defense mechanisms against salinity. Therefore, the evaluation of these traits can help in a better understanding of *S. europaea* adaptive response. Thus, we measured activities of two antioxidant enzymes (CAT and POD) and the concentration of proline as an osmolyte accumulation marker. We found that with increasing NaCl concentration, CAT activity decreases. The highest activity of catalase was recorded in plants growing without salinity (36.7 μM min^−1^ mg^−1^ protein), and the lowest in 1000 mM NaCl (5.7 μM min^−1^ mg^−1^ protein) (Figure 7b). These results suggested that catalase in *S. europaea* is sensitive to salt concentration and in low salinity has high activity and efficiency. In contrast, POD activity (Figure 7c) increased with increasing salinity. The lowest POD activity was in plants treated with 0 mM NaCl (3.7 μM min^−1^ mg^−1^ protein), and the highest activity in 1000 mM NaCl (39.2 μM min^−1^ mg^−1^ protein). In the case of proline, we found any statistically significant differences among all salinity levels (Figure 7d). The proline content in plant tissues ranged from ca. 0.4 μmol/g FW in non-saline conditions to 0.7 μmol/g FW in 1000 mM NaCl. The results suggested that *S. europaea* without NaCl in the medium had a stable proline concentration in tissue which helped it to cope with osmotic stress induced by soil salinity.

## 3. Discussion

Halophytes are plants that have evolved to survive under high salinity conditions, and many of them are thought to require salt exposure for better growth [43]. These plants can regulate their energy metabolism under saline conditions [43,44]. Thus, a better understanding of halophyte functional traits adaptations is important not only for understanding the natural salt marsh ecosystem function [45,46], but also for reconsidering salt tolerance in glycophyte plants to better manage salinity problems in the future agriculture [47]. As it was already mentioned, up to our knowledge, there is a lack of research involving salt stress responses of complex traits from different functioning levels. Addressing our research to answer the question if *S. europaea* traits from different scales are controlled by salinity level, we found that almost all of them were salinity dependent. However, we confirmed our first assumption, i.e., that traits are affected in different ways.

Analysis of growth responses expressed by morphological properties and biomass accumulation demonstrated that the extreme saline condition of 1000 mM negatively affected these traits. Moreover, biomass accumulation was also reduced at 0 and 800 mM compared with 200 and 400 mM NaCl. This is in line with research that already reported stimulation of *S. europaea* and other halophytes biomass accumulation at moderate salinity [48,49]. Research by Lv et al. [28] demonstrated limitation of plant height, stem diameter, and plant biomass in lower salinity than recorded by us, i.e., of ca. 700 mM NaCl. On the other hand, in line with our findings, they also proved *S. europaea* growth traits limitations in the substrate without salinity. Similar results were obtained by Piernik [27], who reported lower *S. europaea* height and biomass at a very low salinity of ca. 2 dSm^−1^ (ca. 20 mM NaCl) in field experiment. However, in this treatment, opposite to our findings, higher number of branches were noted. Additionally, the results of field research by Silva et al. [50] demonstrated a higher number of *S. europaea* branches at low salinity. It is worth emphasizing that morphological parameters under field conditions can be affected not only by salinity but also by plant density and competition between individuals [51]. Obtained results may suggest that plant biomass is more directly salt stress-dependent than morphological traits.

In the present study, an analysis of the anatomical traits demonstrated that the *S. europaea* stem-cortex cells under non-saline conditions and in 1000 mM NaCl were significantly smaller than in other salt treatments. However, we observed that under control conditions, plant cells were more elongated, while in higher salinities, they were rounder. We did not find statistically significant differences in most anatomical traits between plants grown in 200–800 mM NaCl. Our results are in line with other studies on halophytes that show the increasing cells size in water-absorbing tissues under moderately saline conditions [16]. The water storage in the parenchyma is the first *S. europaea* mechanism to overcome high Na^+^ concentrations. This dilutes the accumulated salts and contributes to maintaining cellular turgor, allowing the plant to cope efficiently with high salinity [12,16]. In addition, it was reported that salinity induces vacuolization in many halophytes and may be responsible for the plant adaptation to salt stress [52]. For example, Akcin et al. [52] found that under stressful conditions the juice of halophytes increases. This response is associated with an increase in cell volume due to extra water being stored in the vacuoles for the plant to survive. Moreover, it was reported that *S. europaea* cells can remain turgid and continue proper cell function by ion compartmentalization in cell vacuoles. In this sense, the cytoplasm and organelles of the cell are protected from salt [12]. That is why our results also proved that *S. europaea*, by enlarging its cells under moderately saline conditions, can adapt to high salt stress. On the other hand, the reduction of cell size at extreme salinities can be explained by reduced ability to osmoregulate because of saturation of the system with Na^+^ and Cl^−^ or deficiency of Ca^2+^ and K^+^, which are involved in almost all reactions related to plant development [2].

Our results showed that salinity also affects biochemical traits of *S. europaea*. First of all it negatively affects photosynthetic pigments. We observed that the highest concentration of photosynthetic pigments was under non-saline conditions, and the lowest under the highest salinity level (1000 mM NaCl). These results suggested that salinity significantly affects traits which are responsible for photosynthesis performance in plants. Reduction in photosynthetic pigments due to salt stress has been well documented in numerous papers [5,53,54]. This happens by the inactivation of enzymes involved in the synthesis of photosynthetic pigments [55,56]. Furthermore, this reduction can be due to ROS generation and increasing of chlorophyllase enzyme activity [56,57]. However, we found that photosynthetic pigment concentrations were relatively stable at levels above 400 mM NaCl, indicating that the plant can protect, to a certain extent, these traits under extreme saline conditions. High content of photosynthetic pigments in treatments without salinity was not related to high biomass accumulation, which suggests mechanisms of growth limitations other than those directly related to photosynthetic ability. We also calculated Chlorophyll a/b ratio to test if the rearrangement of chlorophyll contents can explain observed by the pattern in *S. europaea* growth performance, as it was reported, for example, in *Arthrocnemum macrostachyum* and *Sarcocornia fruticosa* by Ghanem et al. [58] (Supplementary Figure S1). Based on the obtained results, we can conclude that such a rearrangement was not a strategy of the investigated species.

We also observed that salinity negatively affects soluble protein content of *S. europaea*. This response may be due to the toxic effect of NaCl on protein synthesis, or the proteolysis of proteins caused by proteases induced by salt stress [59]. It is well documented that high concentrations of NaCl destroy the hydration layer of protein, causing its aggregation and denaturation [60]. However, soluble protein compounds were more stable than pigments and maintained a similar level up to 400 mM NaCl.

Hydrogen peroxide, as already stated, is one of the reactive oxygen species responsible for oxidative stress. On the other hand, H_2_O_2_ is widely generated in many biological systems and mediates various physiological and biochemical processes in plants [61]. Our results showed that the H_2_O_2_ content was the lowest in the control condition, and by increasing salt levels up to 800 mM NaCl, no significant increase was observed. Only at 1000 mM NaCl was its level significantly higher. The opposite pattern, i.e., increasing level of H_2_O_2_ in plant tissues together with increasing salinity, has been observed for glycophytic species [54,62,63]. It seems that halophytic *S. europaea* can prevent H_2_O_2_ overproduction. Nevertheless, in extreme saline conditions, impairment of H_2_O_2_ production and scavenging can affect membrane structural integrity and peroxidation of lipids and limits plants growth and development [19].

In present study, we also monitored traits related to antioxidant enzymes activities and proline content changes. We found that the highest activity of catalase was under non-saline conditions and decreased together with increasing salt levels. Lower activity of CAT in high salt concentrations indicated CAT as a less effective scavenger of H_2_O_2_. Furthermore, the CAT has a poor affinity for H_2_O_2_ and exhibits photo-inactivation and subsequent degradation [64,65]. A completely opposite activity had POD with the lowest values at 0 mM, and the highest at 1000 mM NaCl. In addition, up to 400 mM NaCl POD activity was relatively stable. Increase of the activity of POD at higher salinity levels indicated that this enzyme plays a key role in defense mechanisms of *S. europaea* by scavenging ROS from cells.

Although it is reported that accumulation of proline in *S. europaea* cells is an important adaptive response to salinity [18,43,66], in this study we did not prove a statistically significant increase in proline content by increasing salt levels. Some halophytes produce proline analogues, e.g., glycine betaine under salt stress to survive due to their ability to protect the protein turnover machinery, stabilize proteins, and prevent enzymes from denaturation [66,67]. It seems that *S. europaea* normally has stable proline levels in tissues, which helps it to cope with osmotic stress. However, as proved by Pellegrini et al. [68], proline could be involved in stress tolerance in the *Salicornia* genus regardless of the intensity and duration of the stress. For glycophytic species, a frequently increasing level of proline is observed together with increasing salinity [22,54].

We also referred biochemical traits to dry weight instead of fresh weight to test if differences observed between samples in terms of FW could be partly due to differential loss of water under stress (Supplementary Figures S1 and S2). The tendency in response to salinity level was maintained, and therefore we can conclude that the differences between samples are due to the different levels of stress to which they were subjected.

**Figure 8:**
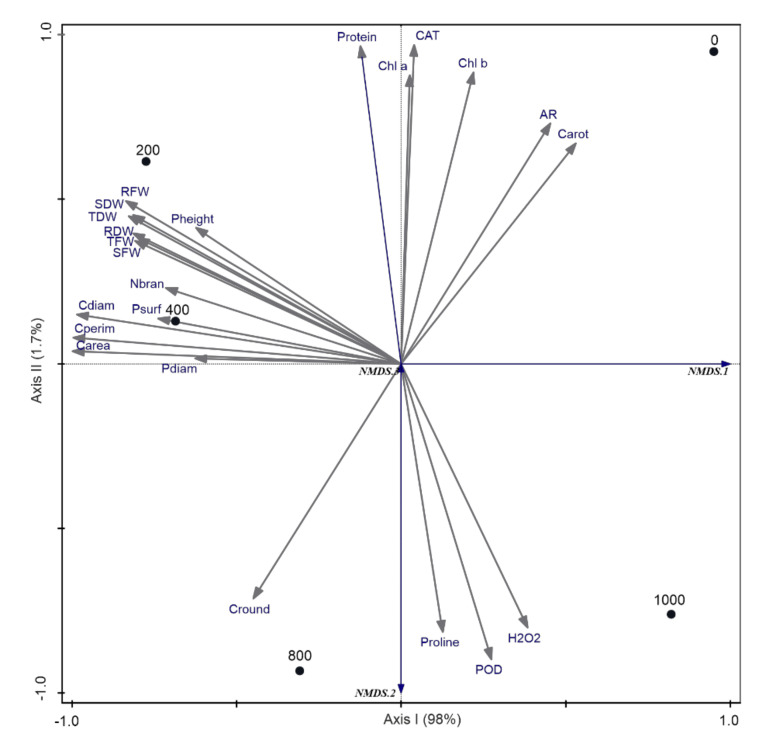
Non-metric multidimentional scaling (NMDS) results—comparison between treatments based on Bray-Curtis dissimilarity measure. Plant functional traits projected as supplementary. Functional traits: Chl a—chlorophyll a, Carot—carotenoids, Psurf—plant surface area, Protein—soluble protein content, POD—peroxidase activity, Nbran—number of branches, AR—aspect ratio, Cperim—cell perimeter, Proline—proline conten, CAT—catalase activity, H_2_O_2_—hydrogen peroxide, Cdiam—cell diameter, Sdiam—shoot diameter, Carea—cell area, SFW—shoot fresh weight, TDW—total dry weight, Pheight—plant height, Chl b—chlorophyll b, RFW—root fresh weight, SDW—shoot dry weight, Cround—cell roundness. Treatments are marked by dots: 0–1000 mM NaCl. Stress = 0.

To conclude on salinity impact, based on all investigated traits and to test our second assumption about indication of plant optimum growth, we performed non-metric multidimensional scaling (NMDS) with Bray-Curtis dissimilarity measure between treatments. Within the analysis we asked for original variables related to the resulting NMDS space as supplementary ones to make the interpretation of the results more clear (Figure 8). Supplementary response variables do not affect the definition of the ordination NMDS axes determined by similarity between samples and can be added to an existing ordination by projection, i.e., by regressing its data on the existing ordination axes [69]. NMDS results demonstrated that some traits can indicate the best *S. europaea* growth expressed by morphological, biomass, and anatomical properties between 200 and 400 mM NaCl (Figure 8). The role of morphological traits adaptations were not stressed up to now. Plants grown in 200 and 400 mM NaCl were the most similar to each other. The first ordination axis explained 98% of the variability between these treatments located at the left side of the diagram and 0 and 1000 mM NaCl located at the right side (Figure 8). The second ordination axis related to the differences between plant traits in 0 and 1000 mM NaCl explained only 1.7% of traits variability, related mostly to oxidative stress. This findings demonstrated by morphological and biomass traits are in line with the field studies of Piernik [27] and the laboratory observations of Lv et al. [28], Cárdenas-Pérez et al. [5], Muscolo et al. [2], and Rozema and Schatb [70] who reported *S. europaea*’s optimum growth at moderate salinity and growth limitation under non-saline conditions. Based on field research and soil sampling on inland salt marshes in Central Europe, Piernik [71] reported the optimum growth of *S. europaea* at 38.1 dSm^−1^, which corresponds to ca. 380 mM NaCl and is in the range of our findings. The growth stimulation at low and moderate salinity in halophytes such as *S. europaea* may be attributed to the improvement in shoot osmotic status because of increased ion uptake [72]. However, the very high salinity imposed a reduction of the growth, which is probably associated with the reduced turgor and the high energy cost of massive salt secretion and osmoregulation [20,48,73].

Based on discriminant analysis with a forward selection procedure and Monte Carlo permutation test, we selected the most affected traits by salinity. There were chlorophyll a, carotenoids, and protein content explaining ca. 23–25% of the variability between all treatments (Table 1), with the highest values related to low saline conditions. A similar amount of variability, i.e., ca. 25% was explained by plant surface area related to higher salinities. Photosynthetic area, represented in *S. europaea* by plant surface area, determines light interception and is an important parameter in determining plant productivity [74]. Of lower importance but statistically significant in the separation between treatments were POD activity related to the highest salinities, explaining ca. 0.8% of variability, and aspect ratio related to non-saline treatment, explaining 0.7%, and the number of branches and cell perimeter related to the moderate salinities, explaining respectively 0.6% and 0.2% of variability between treatments (Table 1). However, as was already mentioned and discussed, the number of branches can be strongly modified under field conditions not only by salinity but also by competition between individuals for resources [51]. We focused here on conditional effects, which exclude the effect of the most correlated traits. Conditional effects summarize the partial effect of each predictor, representing the variation (and its significance) explained by a predictor after accounting for the effect of predictors already selected [69]. The predictors were chosen in the order of their decreasing explained variation. We skipped simple effects, which summarize the independent effects of all explanatory variables, because they reflected significance assessed by already presented ANOVA.

Our results demonstrated that though photosynthetic traits are the most affected by salinity, it does not guarantee high productivity of *S. europaea* in non-saline environments and does not affect productivity to the certain salinity level. This can explain why many halophytes are known as light-required species [75]—because they cope with reduced photosynthetic pigments under saline condition. Fortunately, a relatively higher *S. europaea* area under moderate salinity is advantageous for increasing photosynthetic capacity through capturing light at the expense of increased construction costs and produces large boundary layers responsible for reducing transpiration rates and thus heat exchange and carbon dioxide diffusion to the surrounding air [76]. Important findings in our research are also addressed to the anatomic traits most affected by salinity and related to cell shape. As proved by aspect ratio with increasing salinity, cells become rounder, optimizing their surface area and, as was evidenced by cell perimeter, they become enlarged. However, all regulatory mechanisms behind cell adaptations are still not recognized [21]. Our findings can be the key starting points for their future identification.

Finally, we would like to highlight that *S. europaea* is a species covering different ecotypes/subspecies, which can be specifically adapted to the local environments [5,12,21]. That is why we reported, in the introduction, *Salicornia ramosissima* J. Woods (= *S. herbacea* L.) from Central Europe [9] as a synonym/reference for the *S. europaea* population investigated by us.

## 4. Materials and Methods

### 4.1. Laboratory Plant Material and Plantlets Preparation

*S. europaea* seeds were collected from the nature reserve of halophytes “Ciechocinek” located in north-central Poland (52°53′ N, 18°47′ E; Central Europe) under permission WOP.6400.12.2020.JC. Matured plants spikes were harvested at the beginning of November, and the seeds were shaken from the spikes. After threshing, 250 seeds that were healthy and uniform in appearance were selected and planted in Petri dishes with Whatman no. 2 filter paper. The Petri dishes were watered with 5 mL distilled water and were placed in a growth chamber with 22 °C and 16 h light period for 9 days. After full germination, uniform plantlets were selected and planted into the pots that contained a mixture of vermiculite and sand (1:1). In total, 60 pots were prepared and divided into five groups (12 pots per each salt concentrations: 0 mM—distilled water, 200 mM, 400 mM, 800 mM, and 1000 mM NaCl). Before planting, each group of 12 pots was located on individual tray lacking drainage and were saturated to their full capacity with relevant NaCl solution (ca. 0.5 L per treatment, pot size: height 5.3 cm, diameter 5.5 cm). All pots were placed in a growth chamber with 22 °C and a 16 h light period and each group was irrigated through pouring distillate water once per week. To prevent nutrient deficiencies, plants were watered with Hoagland’s solution once per two weeks [77]. In our previous research we focused on germination and early growth assessments of *S. europaea* [2]. Therefore, this time we focused on plantlets, which were grown for 60 days to the end of the experiment period.

### 4.2. Growth Analysis

Growth analysis covered morphological and biomass assessments. Two months after starting salt treatments, the plants were subjected to morphological analysis. For this purpose, plant images were taken by a Canon camera with resolution of 5 to 25 MP for morphological analysis. Images were taken from four sides of each sample (see Figure 1) in four replications. We used ImageJ version 1.47 program (National Institutes of Health, Bethesda, MD, USA) for image analysis to measure plant morphological parameters: height, number of branches, surface area, and shoot diameter. The results were calculated according to the number of pixels covering the plant converted to the appropriate metric units.

At the end of the experiment, shoot fresh weight (SFW), root fresh weight (RFW), and total fresh weight (TFW) were measured. After the samples were oven dried for 72 h at 72 °C, shoot dry weight (SDW), root dry weight (RDW), and total dry weight (TDW) were determined.

### 4.3. Anatomical Analysis

The anatomical analysis was performed based on the cross-sections of plant shoots (middle primary branch—fleshy segment shoot) taken from plants growing in five NaCl concentrations. Slices of fresh tissue were obtained by cutting them with a sharp bi-shave blade and slices of approximately 0.5 mm were used for analysis. The samples were stained with alkaline fuchsin and malachite green for microscopic observations. *S. europaea* cross-sections photos for each NaCl concentration were taken using light microscopy. Photos were used to observe the cortex parenchyma cells, because water storage in the parenchyma is the first *S. europaea* mechanism to overcome high Na^+^ concentrations [28]. We used ImageJ version 1.47 (National Institutes of Health, Bethesda, MD, USA) for image analysis. For each sample, more than 200 cells were analyzed to determine the following parameters: (1) cell area (A), which was calculated through the number of pixels inside the borderline; (2) cell perimeter, which was calculated due to the prescribed limits; (3) cell diameter, which was defined by the distance between two points; (4) aspect ratio (AR), which was defined as the quotient between the minimum and maximum diameter, determining the uniformity of the cell; and (5) cell roundness (R), which determines the circularness of a cell using Equation (1). In this equation, for perfectly round cells R = 1 [78].

R = (4A)/(π(MD)^2^), (1)

where: A is cell area; MD is cell diameter.

### 4.4. Biochemical Analyzes

To evaluate the effect of different salinity levels on the biochemical traits of *S. europaea* plants, we measured traits related to photosynthetic activity, osmotic adjustment, oxidative stress, and enzymatic activities.

#### 4.4.1. Photosynthetic Pigments Content

Chlorophylls (Ch a and Ch b) and carotenoids were extracted from fresh plant stems (100 mg) using 80% acetone for 6 h in darkness, and then centrifuged at 10,000 rpm, 10 min. Supernatants were quantified spectrophotometrically. The absorbances at 646, 663, and 470 nm wavelength were measured. The total content of chlorophyll a and b [79] and carotenoids [80], when 80% of acetone was used as dissolvent, were calculated according to Equations (2)–(4) and reported as mg-per-gram fresh weight.

Chlorophyll (a) = [(12.21 × Abs663) − (2.81 × Abs646) × mLAcetone]/mgSteam(2)

Chlorophyll (b) = [(20.13 × Abs646) − (2.81 × Abs663) × mLAcetone]/mgSteam(3)

Carotenoids = (([(1000 × Abs470 − 3.27(Chla) − 104(Chlb))/227] × mLAcetone))/mgSteam(4)

#### 4.4.2. Soluble Protein Content

Plant material (0.5 g) was placed in a mortar chilled with liquid nitrogen, then 7 mL of 50 mM phosphate buffer was added and homogenized. After fine grinding, the sample was centrifuged at 20,000× *g* for 25 min at 4 °C. The supernatant was collected for determination of protein content at 595 nm using the Bradford method [81]. The amount of protein was determined using a bovine serum albumin as a standard.

#### 4.4.3. Hydrogen Peroxide (H_2_O_2_) Content

Hydrogen peroxide was measured according to the methods described by Velikova et al. [82]. Three samples were prepared for each NaCl concentration. Stem tissues (0.5 g) were homogenized in an ice bath with 5 mL of 0.1% TCA (trichloroacetic acid). Then, the homogenate was centrifuged at 12,000× *g* for 15 min at 4 °C. The supernatant (0.5 mL) was transferred to a new tube and 0.5 mL of 10 mM potassium phosphate (pH 7) and 2 mL of 1 M KI were added. The solution was incubated in the dark for one hour and then the absorbance was read at 390 nm wavelength and the hydrogen peroxide concentration was given based on the standard curve from 0 to 40 nM and equation y = 0.0188x + 0.046, R^2^ = 0.987 in nM-per-gram fresh weight.

#### 4.4.4. Peroxidase (POD) Activity

The peroxidase activity was measured according to the method of Chance and Maehly [83]. For the measurement, a 3-mL reaction mixture was prepared with the following composition: 50 mM potassium phosphate buffer (pH 7), 20 mM guaiacol, 40 mM H_2_O_2_, and 0.1 mL of enzyme extract obtained from plant stems. The reaction was initiated by adding the enzyme extract. The increase in absorbance of the reaction solution was measured at 470 nm wavelength. The reading was recorded every 20 s. One unit of peroxidase activity was defined as an absorbance change of 0.01 units per minute. The enzyme activity was expressed on a protein basis [84].

#### 4.4.5. Catalase (CAT) Activity

Catalase was determined by measuring residual H_2_O_2_ with a titanium reagent [85]. The reaction mixture was 3 mL in total and consisted of: 1 mL of 6 mM H_2_O_2_ and 1.9 mL of 0.1 M phosphate buffer (pH 7). To start the reaction, 0.1 mL of diluted enzyme extract was added to the test tube. The reaction was stopped after 5 min by adding 4 mL of titanium reagent, which formed a colored complex with residual H_2_O_2_. Mixtures without enzyme were used as control. The colored complex was centrifuged at 10,000× *g* for 10 min. The absorbance was measured at 415 nm. The residual H_2_O_2_ content was calculated from the standard curve [86].

#### 4.4.6. Proline Content

The proline content of the leaves was determined according to the method of Bates et al. [87]. Three samples were prepared for each NaCl concentration; 0.5 g of each sample were pulverized on ice and homogenized in a mortar with 5 mL of 3% aqueous sulfosalicylic acid. The homogenate was centrifuged at 18,000× *g*, 10 min at 4 °C, and the supernatant was collected. About 1 mL of the supernatant was added into a test tube and 2 mL of glacial acetic acid, 2 mL of ninhydrin reagent, and 1 mL of sulfosalicylic acid 3% were added to the tube. The reaction mixture was boiled in a 100 °C water bath for one hour. Then, the test tubes were put on ice and 4 mL of toluene were added to them. The samples were transferred to a separating funnel and after thorough mixing, the toluene containing the chromophore was separated. The absorbances were measured at 520 nm wavelength. The amount of proline was determined using the standard curve in the concentration range of 0 to 40 µg mL^−1^ and equation y = 0.0467x − 0.0734, R^2^ = 0.963.

### 4.5. Statistical Analyzes

To compare all treatments, one-way analysis of variance (ANOVA) was performed followed by post hoc analysis using Tukey’s test. The PAST 4.0 program [88] was used for statistical analyses. To discuss *S. europaea*’s optimum growth based on the whole set of the traits, we applied non-metric multidimensional scaling (NMDS) with Bray-Curtis dissimilarity measure to demonstrate similarities between plants coming from different salinity treatments. To select the most affected traits by salinity treatments, we applied discriminant analysis with a forward-selection procedure and Monte Carlo permutation test. Only conditional effects were taken into account to exclude the effect of the most correlated traits [42]. For these analyses, the Canoco 5.0 program was applied [69].

## 5. Conclusions

Addressing our research to answer the question if *S. europaea* traits from different scales are controlled by salinity level, we found that almost all of them were salinity dependent. However, we proved that functional traits were affected by salinity in the different ways and demonstrated significant differences at different salinity levels. Moreover, we did not find a statistically significant relationship between proline levels and increasing salinity, which was not expected based on reported findings regarding glycophyte species. Based on analysis of all investigated traits, we demonstrated that morphological, biomass, and anatomical properties indicated optimum growth between 200 and 400 mM NaCl and growth limitations at 0, 800, 1000 mM NaCl. Moreover, we can conclude that the most affected traits by salinity include photosynthetic pigments, protein content, plant surface area, peroxidase activity, and anatomic traits related to cell shape. Our results significantly expand the knowledge about *S. europaea* functional traits variation in response to salinity, which can be important for discovering regulating processes and for possible future agricultural applications.

## Figures and Tables

**Table 1:** Results of discriminant analysis (CVA) with forward selection and Monte Carlo permutation test demonstrating relative importance and statistical significance of plant traits in the separation of salt treatments 0, 200, 400, 800, and 1000 mM NaCl. Multi-correlated traits have been automatically excluded.

Conditional Effects			
Variable	V %	Pseudo-F	*p*
Chl a	24.8	7.2	0.002
Carot	24.7	10.3	0.002
Psurf	24.5	18.8	0.002
Protein	23.1	147	0.002
POD	0.8	6.5	0.014
Nbran	0.6	6.4	0.012
AR	0.7	11.3	0.004
Cperim	0.2	3.9	0.03
Proline	<0.1	1	0.372
CAT	<0.1	1.4	0.286
H_2_O_2_	<0.1	0.8	0.516
Cdiam	<0.1	1.3	0.304
Sdiam	<0.1	0.6	0.598
Carea	<0.1	1	0.396
SFW	<0.1	0.9	0.452
TDW	<0.1	1.2	0.334
Pheight	<0.1	0.6	0.624
Chl b	<0.1	2.2	0.144
RFW	<0.1	0.7	0.5
SDW	<0.1	0.8	0.466
Cround	<0.1	0.2	0.852

Functional traits: Chl a—chlorophyll a, Carot—carotenoids, Psurf—plant surface area, Protein—soluble protein content, POD—peroxidase activity, Nbran—number of branches, AR—aspect ratio, Cperim—cell perimeter, Proline—proline conten, CAT—catalase activity, H_2_O_2_—hydrogen peroxide, Cdiam—cell diameter, Sdiam—shoot diameter, Carea—cell area, SFW—shoot fresh weight, TDW—total dry weight, Pheight—plant height, Chl b—chlorophyll b, RFW—root fresh weight, SDW—shoot dry weight, Cround—cell roundness. V—variability.

## Data Availability

The data presented in this study are available on request from the corresponding authors. The data are not publicly available due to ongoing research.

## Supplementary Materials

The following supporting information can be downloaded at: https://www.mdpi.com/article/10.3390/plants11081051/s1, Figure S1: The average concentration of: (a) chlorophyll a; (b) chlorophyll b; (c) chlorophyll a/b ratio, (d) carotenoid and (e) soluble proteins in the tested plants; Figure S2: The average parameters of: (a) H_2_O_2_ content; (b) proline content in the tested plants.

## References

[1] Santos J., Al-Azzawi M., Aronson J., Flowers T.J. (2016). eHALOPH a database of salt-tolerant plants: Helping put halophytes to work. Plant Cell Physiol..

[2] Muscolo A., Panuccio M.R., Piernik A., Khan M.A., Böer B., Öztürk M., Al Abdessalaam T.Z., Clüsener-Godt M., Gul B. (2014). Ecology, Distribution and Ecophysiology of *Salicornia Europaea* L.. Sabkha Ecosystems.

[3] Kadereit G., Ball P., Beer S., Mucina L., Sokoloff D., Teege P., Yaprak A.E., Freitag H. (2007). A taxonomic nightmare comes true: Phylogeny and biogeography of glassworts (*Salicornia* L., *Chenopodiaceae*). Taxon.

[4] Cárdenas-Pérez S., Chanona-Pérez J.J., de Jesús Perea-Flores M., Calderon H., Piernik A., López-Soto K.D., González C.B.G. (2020). Microstructure of *Salicornia bigelovii* stems under photonic and electron microscopy. Microsc. Microanal..

[5] Cárdenas-Pérez S., Piernik A., Ludwiczak A., Duszyn M., Szmidt-Jaworska A., Chanona-Pérez J. (2020). Image and fractal analysis as a tool for evaluating salinity growth response between two *Salicornia europaea* populations. BMC Plant Biol..

[6] Negrão S., Schmöckel S., Tester M. (2017). Evaluating physiological responses of plants to salinity stress. Ann. Bot..

[7] Loconsole D., Cristiano G., De Lucia B. (2019). Glassworts: From wild salt marsh species to sustainable edible crops. Agriculture.

[8] Araus J.L., Rezzouk F.Z., Thushar S., Shahid M., Elouafi I.A., Bort J., Serret M.D. (2021). Effect of irrigation salinity and ecotype on the growth, physiological indicators and seed yield and quality of *Salicornia europaea*. Plant. Sci..

[9] Krüger A., Hellwig F., Oberprieler C. (2002). Genetic diversity in natural and anthropogenic inland populations of salt-tolerant plants: Random amplified polymorphic DNA analyses of *Aster tripolium* L. (*Compositae*) and *Salicornia ramosissima* Woods (*Chenopodiaceae*). Mol. Ecol..

[10] Antunes M.D., Gago C., Guerreiro A., Sousa A.R., Julião M., Miguel M.G., Faleiro M.L., Panagopoulos T. (2021). Nutritional characterization and storage ability of *Salicornia ramosissima* and *Sarcocornia perennis* for fresh vegetable salads. Horticulturae.

[11] Rhee M.H., Park H.-J., Cho J.Y. (2009). *Salicornia herbacea*: Botanical, chemical and pharmacological review of halophyte marsh plant. J. Med. Plants Res..

[12] Cárdenas-Pérez S., Piernik A., Chanona-Pérez J.J., Grigore M.N., Perea-Flores M.J. (2021). An overview of the emerging trends of the *Salicornia* L. genus as a sustainable crop. Environ. Exp. Bot..

[13] Liu X.G., Xia Y.G., Wang F., Sun M., Jin Z.J., Wang G.T. (2005). Analysis of fatty acid composition of *Salicornia europaea* L. seed oil. Food Sci..

[14] Gunning D. (2016). Cultivating Salicornia Europaea (Marsh Samphire).

[15] Patel S. (2016). Salicornia: Evaluating the halophytic extremophile as a food and a pharmaceutical candidate. 3 Biotech.

[16] Grigore M.-N., Toma C. (2021). Morphological and anatomical adaptations of halophytes: A review. Handbook of Halophytes: From Molecules to Ecosystems towards Biosaline Agriculture.

[17] Flowers T.J., Colmer T.D. (2008). Salinity tolerance in halophytes. New Phytol..

[18] Flowers T.J., Hajibagher M.A., Yeo A.R. (1991). Ion accumulation in the cell walls of rice plants growing under saline conditions: Evidence for the Oertli hypothesis. Plant Cell Environ..

[19] Aghaleh M., Niknam V., Ebrahimzadeh H., Razavi K. (2011). Effect of salt stress on physiological and antioxidative responses in two species of Salicornia (*S. persica* and *S. europaea*). Acta Physiol. Plant..

[20] Mohammadi H., Kardan J. (2016). Morphological and physiological responses of some halophytes to salinity stress. Ann. Univ. Mariae Curie Sklodowska Sect. C Biol..

[21] Cárdenas-Pérez S., Niedojadło K., Mierek-Adamska A., Dąbrowska G.B., Piernik A. (2022). Maternal salinity influences anatomical parameters, pectin content, biochemical and genetic modifications of two *Salicornia europaea* populations under salt stress. Sci. Rep..

[22] Mohamed M., El-Maboud A. (2021). Seasonal Physiological Response of *Salicornia Europaea* L.. Res. Rev. J. Bot. Sci..

[23] Akcin A., Yalcin E. (2016). Effect of salinity stress on chlorophyll, carotenoid content, and proline in *Salicornia prostrata* Pall. and *Suaeda prostrata* Pall. subsp. *prostrata* (Amaranthaceae). Braz. J. Bot..

[24] Rodríguez-Álvarez M., Ledea-Rodríguez J.L., Murillo-Amador B., Mazón-Suástegui J.M. (2022). Morphometry and chlorophyll content of *Salicornia bigelovii* (*Torr*) under sea water treatments and *Natrum muriaticum* as a saline stress mitigator. Trop. Subtrop. Agroecosystems.

[25] Wang X., Fan P., Song H., Chen X., Li X., Li Y. (2009). Comparative proteomic analysis of differentially expressed proteins in shoots of *Salicornia europaea* under different salinity. J. Proteome Res..

[26] Snow A.A., Vince S.W. (1984). Plant zonation in Alaskan salt-marsh. II. An Experimental study of the role of edaphic conditions. J. Ecol..

[27] Piernik A. (2006). Growth of three meadow species along a salinity gradient in an inland saline habitat: Transplant experiment. Pol. J. Ecol..

[28] Lv S., Jiang P., Chen X., Fan P., Wang X., Li Y. (2012). Multiple compartmentalization of sodium conferred salt tolerance in *Salicornia europaea*. Plant Physiol. Biochem..

[29] Singh D., Buhmann A.K., Flowers T.J., Seal C.E., Papenbrock J. (2014). Salicornia as a crop plant in temperate regions: Selection of genetically characterized ecotypes and optimization of their cultivation conditions. AoB Plants.

[30] Rao G.M.N., Murty P.P. (2013). Morphological and anatomical features of *Salicornia brachiata Roxb*. J. Biol. Chem. Res..

[31] Violle C., Navas M.L., Vile D., Kazakou E., Fortunel C., Hummel I., Garnier E. (2007). Let the concept of trait be functional!. Oikos.

[32] Chapin F.S., Zavaleta E.S., Eviner V.T., Naylor R.L., Vitousek P.M., Reynolds H.L., Hooper D.U., Lavorel S., Sala O.E., Hobbie S.E. (2000). Consequences of changing biodiversity. Nature.

[33] Enquist B.J., Condit R., Peet R.K., Schildhauer M., Thiers B.M. (2016). Cyberinfrastructure for an integrated botanical information network to investigate the ecological impacts of global climate change on plant biodiversity. PeerJ Prepr..

[34] Kattge J., Bönisch G., Dìaz S., Lavorel S., Prentice I.C., Leadley P., Tautenhahn S., Werner G.D.A., Aakala T., Abedi M. (2020). TRY plant trait database—Enhanced coverage and open access. Glob. Chang. Biol..

[35] Zirbel C.R., Bassett T., Grmanand E., Brudvig L.A. (2017). Plant functional traits and environmental conditions shape community assembly and ecosystem functioning during restoration. J. Appl. Ecol..

[36] Bu W., Huang J., Xu H., Zang R., Ding Y., Li Y., Lin M., Wang J., Zhang C. (2019). Plant Functional Traits Are the Mediators in Regulating Effects of Abiotic Site Conditions on Aboveground Carbon Stock-Evidence from a 30 ha Tropical Forest. Plot. Front. Plant Sci..

[37] Helsen K., Acharya K.P., Brunet J., Cousins S.A.O., Decocq G., Kolb A., Lemke I.H., Lenoir J., Plue J., Verheyen K. (2017). Biotic and abiotic drivers of intraspecific trait variation within plant populations of three herbaceous plant species along a latitudinal gradient. BMC Ecol..

[38] Pérez-Harguindeguy N., Díaz S., Garnier E., Lavorel S., Poorter H., Jaureguiberry P., Cornelisson J.H.C. (2013). New handbook for standardised measurement of plant functional traits worldwide. Aust. J. Bot..

[39] Raffard A., Lecerf A., Cote J., Buoro M., Lassus R., Cucherousset J. (2017). The functional syndrome: Linking individual trait variability to ecosystem functioning. Proc. R. Soc. B Biol. Sci..

[40] Fan P., Nie L., Jiang P., Feng J., Lv S., Chen X., Bao H., Guo J., Tai F., Wang J. (2013). Transcriptome Analysis of *Salicornia europaea* under Saline Conditions Revealed the Adaptive Primary Metabolic Pathways as Early Events to Facilitate Salt Adaptation. PLoS ONE.

[41] Kaleem M., Hameed M. (2021). Functional traits for salinity tolerance in differently adapted populations of *Fimbristylis complanata* (Retz.). Int. J. Phytoremediation.

[42] Šmilauer P., Lepš J. (2014). Multivariate Analysis of Ecological Data using CANOCO 5.

[43] Kumar A., Mann A., Lata C., Kumar N., Sharma P. (2019). Salinity-induced physiological and molecular responses of halophytes. Research Developments in Saline Agriculture.

[44] Winicov I., Bastola D.R. (1997). Salt tolerance in crop plants: New approaches through tissue culture and gene regulation. Acta Physiol. Plant..

[45] Ulrich W., Kubota Y., Piernik A., Gotelli N.J. (2018). Functional traits and environmental characteristics drive the degree of competitive intransitivity in European saltmarsh plant communities. J. Ecol..

[46] Ulrich W., Hulisz P., Mantilla-Contreras J., Elvisto T., Piernik A. (2019). Compensatory effects stabilize the functioning of Baltic brackish and salt marsh plant communities. Estuar. Coast. Shelf Sci..

[47] Shabala S. (2013). Learning from halophytes: Physiological basis and strategies to improve abiotic stress tolerance in crops. Ann. Bot..

[48] Aghaleh M., Niknam V., Ebrahimzadeh H., Razavi K. (2009). Salt stress effects on growth, pigments, proteins and lipid peroxidation in *Salicornia persica* and *S. europaea*. Biol. Plant..

[49] Orlovsky N., Japakova U., Zhang H., Volis S. (2016). Effect of salinity on seed germination, growth and ion content in dimorphic seeds of *Salicornia europaea* L. (*Chenopodiaceae*). Plant Divers.

[50] Silva H., Caldeira G., Freitas H. (2007). *Salicornia ramosissima* population dynamics and tolerance of salinity. Ecol. Res..

[51] Craine J.M., Dybzinski R. (2013). Mechanisms of plant competition for nutrients, water and light. Funct. Ecol..

[52] Akcin T.A., Akcin A., Yalcın E. (2017). Anatomical changes induced by salinity stress in *Salicornia freitagii* (*Amaranthaceae*). Braz. J. Bot..

[53] Gong D.H., Wang G.Z., Si W.T., Zhou Y., Liu Z., Jia J. (2018). Effects of salt stress on photosynthetic pigments and activity of ribulose-1, 5-bisphosphate carboxylase/oxygenase in *Kalidium foliatum*. Russ. J. Plant Physiol..

[54] Rajabi Dehnavi A., Zahedi M., Ludwiczak A., Piernik A. (2022). Foliar Application of Salicylic Acid Improves Salt Tolerance of Sorghum (*Sorghum bicolor* (L.) Moench). Plants.

[55] Ashraf M. (1989). The effect of NaCl on water relations, chlorophyll, and protein and proline contents of two cultivars of blackgram (*Vigna mungo* L.). Plant Soil.

[56] Arif Y., Singh P., Siddiqui H., Bajguz A., Hayat S. (2020). Salinity induced physiological and biochemical changes in plants: An omic approach towards salt stress tolerance. Plant Physiol. Biochem..

[57] Arulbalachandran D., Ganesh K.S., Subramani A. (2009). Changes in metabolites and antioxidant enzyme activity of three *Vigna* species induced by NaCl stress. Am. Eurasian J. Agron..

[58] Ghanem A.F.M., Mohamed E., Kasem A.M.M.A., El-Ghamery A.A. (2021). Differential Salt Tolerance Strategies in Three Halophytes from the Same Ecological Habitat: Augmentation of Antioxidant Enzymes and Compounds. Plants.

[59] Parida A.K., Das A.B., Mittra B., Mohanty P. (2004). Salt-stress induced alterations in protein profile and protease activity in the *mangrove Bruguiera parviflora*. Z. Für Nat. C.

[60] Fogarty A.C., Duboué-Dijon E., Sterpone F., Hynes J.T., Laage D. (2013). Biomolecular hydration dynamics: A jump model perspective. Chem. Soc. Rev..

[61] Li J.-T., Qiu Z.-B., Zhang X.-W., Wang L.-S. (2011). Exogenous hydrogen peroxide can enhance tolerance of wheat seedlings to salt stress. Acta Physiol. Plant..

[62] Liu L., Huang L., Lin X., Sun C. (2020). Hydrogen peroxide alleviates salinity-induced damage through enhancing proline accumulation in wheat seedlings. Plant Cell Rep..

[63] Hernandez M., Fernandez-Garcia N., Diaz-Vivancos P., Olmos E. (2010). A different role for hydrogen peroxide and the antioxidative system under short and long salt stress in *Brassica oleracea* roots. J. Exp. Bot..

[64] Shang W., Feierabend J. (1999). Dependence of catalase photoinactivation in rye leaves on light intensity and quality and characterization of a chloroplast-mediated inactivation in red light. Photosynth. Res..

[65] Amor N.B., Jiménez A., Megdiche W., Lundqvist M., Sevilla F., Abdelly C. (2006). Response of antioxidant systems to NaCl stress in the halophyte *Cakile maritima*. Physiol. Plant..

[66] Moghaieb R.E.A., Saneoka H., Fujita K. (2004). Effect of salinity on osmotic adjustment, glycinebetaine accumulation and the betaine aldehyde dehydrogenase gene expression in two halophytic plants, *Salicornia europaea* and *Suaeda maritima*. Plant Sci..

[67] Shoukat E., Ahmed M.Z., Abideen Z., Azeem M., Ibrahim M., Gul B., Khan M.A. (2020). Short and long term salinity induced differences in growth and tissue specific ion regulation of *Phragmites karka*. Flora.

[68] Pellegrini E., Forlani G., Boscutti F., Casolo V. (2020). Evidence of non-structural carbohydrates-mediated response to flooding and salinity in *Limonium narbonense* and *Salicornia fruticosa*. Aquat. Bot..

[69] Ter Braak C.J.F., Smilauer P. (2012). Canoco Reference Manual and User’s Guide: Software for Ordination, Version 5.0.

[70] Rozema J., Schat H. (2013). Salt tolerance of halophytes, research questions reviewed in the perspective of saline agriculture. Environ. Exp. Bot..

[71] Piernik A. (2012). Ecological Pattern of Inland Salt Marsh Vegetation in Central Europe.

[72] Naidoo G., Naidoo Y. (1998). Salt tolerance in *Sporobolus virginicus*: The importance of ion relations and salt secretion. Flora.

[73] Kong Y., Zheng Y. (2014). Potential of Producing *Salicornia bigelovii* Hydroponically as a Vegetable at Moderate NaCl Salinity. HortScience Horts.

[74] Koester R.P., Skoneczka J.A., Cary T.R., Diers B.W., Ainsworth E.A. (2014). Historical gains in soybean (*Glycine max* Merr.) seed yield are driven by linear increases in light interception, energy conversion, and partitioning efficiencies. J. Exp. Bot..

[75] Ellenberg H., Weber H.E., Düll R., Wirth V., Werner W., Paulißen D. (1992). Indicator values of plants in Central Europe. Scr. Geobot..

[76] Díaz S., Kattge J., Cornelissen J.H.C., Wright I.J., Lavorel S., Dray S., Reu B., Kleyer M., Wirth C., Prentice I.C. (2016). The global spectrum of plant form and function. Nature.

[77] Hoagland D.R., Arnon D.I. (1950). The Water-Culture Method for Growing Plants without Soil. Circular California Agricultural Experiment Station.

[78] Cárdenas-Pérez S., Chanona-Pérez J., Méndez-Méndez J., Calderón-Domínguez G., López-Santiago R., Arzate-Vázquez I. (2016). Nanoindentation study on apple tissue and isolated cells by atomic force microscopy, image and fractal analysis. Innov. Food Sci. Emerg. Technol..

[79] Arnon D.I. (1949). Copper enzymes in isolated chloroplasts. Polyphenoloxidase in *Beta vulgaris*. Plant Physiol..

[80] Lichtenthaler H.K., Wellburn A.R. (1983). Determinations of total carotenoids and chlorophylls a and b of leaf extracts in different solvents. Biochem. Soc. Trans..

[81] Bradford M.M. (1976). A rapid and sensitive method for the quantitation of microgram quantities of protein utilizing the principle of protein-dye binding. Anal. Biochem..

[82] Velikova V., Yordanov I., Edreva A. (2000). Oxidative stress and some antioxidant systems in acid rain-treated bean plants: Protective role of exogenous polyamines. Plant Sci..

[83] Chance B., Maehly A. (1955). The assay of catalases and peroxidases. Methods Biochem. Anal..

[84] Rached-Kanouni M., Alatou D. (2013). Change in activity of antioxidative enzymes in leaves of *Acacia retinodes*, *Biota orientalis* and *Casuarina equisetifolia* under heat stress condition. Eur. Sci. J..

[85] Teranishi Y., Tanaka A., Osumi M., Fukui S. (1974). Catalase activities of hydrocarbon-utilizing *Candida* yeasts. Agric. Biol. Chem..

[86] Sairam R.K., Rao K.V., Srivastava G. (2002). Differential response of wheat genotypes to long term salinity stress in relation to oxidative stress, antioxidant activity and osmolyte concentration. Plant Sci..

[87] Bates L.S., Waldren R.P., Teare I. (1973). Rapid determination of free proline for water-stress studies. Plant Soil.

[88] Hammer Ø., Harper D.A., Ryan P.D. (2001). PAST: Paleontological statistics software package for education and data analysis. Palaeontol. Electron..

