# Supplementary material for: Salicornia europaea L. Functional Traits Indicate Its Optimum Growth
Source: Plants (Basel). 2022 Apr 12;11(8):1051. doi: 10.3390/plants11081051 (PMC9033102; doi:10.3390/plants11081051)
Supplement: Supplementary file 1 [file plants-11-01051-s001.zip › plants-1624006-supplementary.pdf]

Supplementary material

Salicornia europaea L. Functional Traits Indicate Its Optimum Growth

Stefany Cárdenas-Pérez et al.

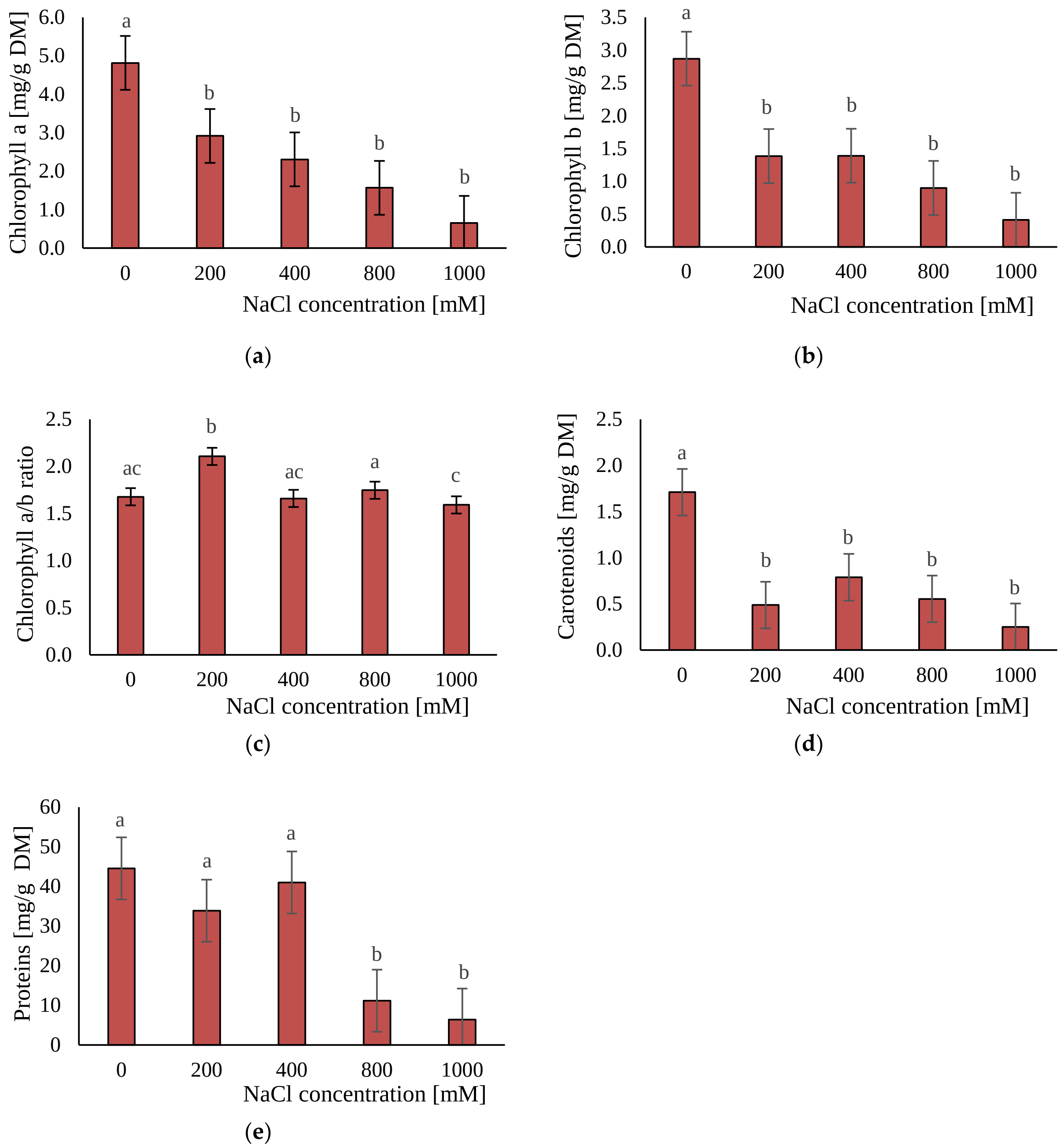

Figure S1. The average concentration of: (**a**) chlorophyll a; (**b**) chlorophyll b; (**c**) chlorophyll a/b ratio, (**d**) carotenoid and (**e**) soluble proteins in the tested plants; ± SD in the tested samples. ANOVA $p < 0.001$, significant differences based on post hoc Tukey's test are marked with different letters. Dry mass (DM) was applied as reference base.

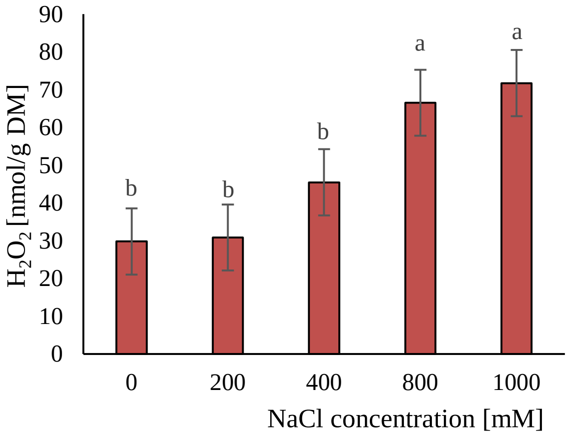

(**a**)

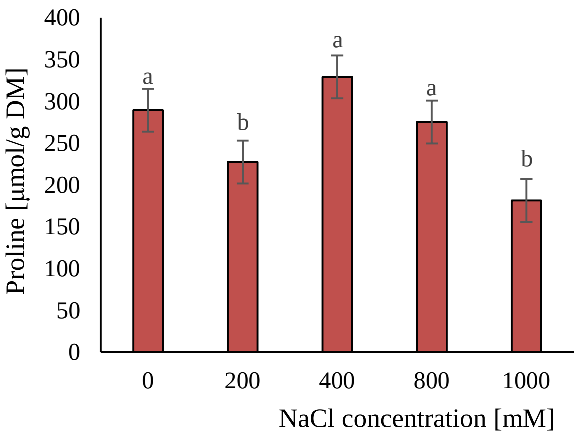

(**b**)

Figure S2. The average parameters of: (**a**) H_2O_2 content; (**b**) proline content in the tested plants; ± SD in the tested samples. ANOVA $p < 0.001$, significant differences based on post hoc Tukey's test are marked with different letters. Dry mass (DM) was applied as reference base.
